# Multi potent aromatic nano colloid: synthesis, characterization and applications

**DOI:** 10.1186/s13568-020-01104-5

**Published:** 2020-09-18

**Authors:** Ranjani Soundhararajan, Salman Al Farzi Mohamed Sheik Meeran, Shruthy Priya Prakash, Waseem Mohammad, Ruckmani Kandasamy, Hemalatha Srinivasan

**Affiliations:** 1 School of Life Sciences, B. S. Abdur Rahman Crescent institute of Science and Technology, Vandalur, 600048 Chennai, India; 2 Department of Pharmaceutical Technology, University College of Engineering, Anna University BIT Campus, Tiruchirappalli, Tamilnadu 620024 India

**Keywords:** Aromatic nanocolloids, CTX-M-15, AmpC, Antibiotic resistance, Fungicide, Breast cancer MCF-7, Anticancer

## Abstract

In this study the aromatic nanocolloids (CANCs) are synthesized using the noble metal silver by using Citronella extract and confirmed through physio chemical analysis. The synthesised CANCs were evaluated for its antimicrobial activity and antibiofilm activity against pathogenic biofilm forming *E. coli*. In addition, synthesized CANCs were evaluated for the expression of virulent genes encoding AmpC and CTX-M-15. The results confirmed that CANCs showed effective antimicrobial activity through its bacteriostatic, bactericidal and quorum quencher activity and downregulated CTX-M-15 gene. CANCs were validated as alternate to the commercial fungicides to control plant pathogenic fungi such as *A. niger* MTCC (281), *Fusarium graminearum* MTCC (2089) and *F. udum* MTCC (2204). Furthermore, analysis of CANCs on breast cancer (MCF-7) cells under in vitro condition showed that the cytotoxicity of CANCs is dose dependent. Thus, the multifunctional CANCs can be utilized as potential antimicrobial, antifungal and anticancer agent.
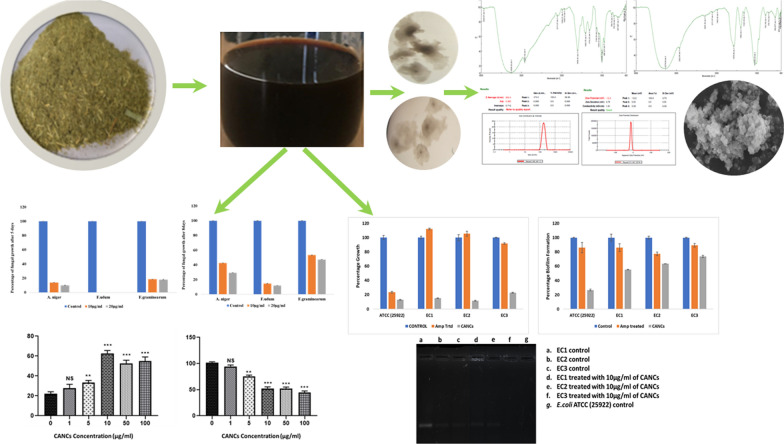

## Introduction

Nanotechnology is the most important upcoming field in material science and technology. Nanocolloids are utilised because of its unique properties such as small size, shape, orientation and compatibility (Iravani et al. 2014). Silver Nanocolloids are widely used in the field of medicine, drug discovery, disease monitoring, disease therapy etc. Several physical and chemical methods are used for the stabilization of green nanocolloids, which are non-toxic to plants and animals and toxic to bacterial cell. Hence, Green nanocolloids are eco-friendly, benign, reliable, and less toxic (Patra et al. 2018).

*Cymbopogon citratus*, which is commonly called as citronella, lemon grass etc., because of its unique lemony fragrance. Aromatic citronella plants can be used as an alternative solution to chemically synthesised compounds (Masurkar et al. 2011). Citronella extracts namely geranyl acetate, citral, neral, monoterpenes, cymene, terpinene, and linalool have been reported to possess several bioactivities such as antibacterial, antifungal and anticancer property.

Biofilm is a group of microorganisms that lives as a community over the solid surfaces in moist environment. Urinary tract infections and intestinal infections are majorly caused by biofilm forming *Escherichia coli.* Biofilm forming *E. coli* pathogens will lead to infections such as wounds, pneumonia, implant, catheter-mediated infections which affect people all around the world (Mittal et al. 2015). Extracellular polymeric substances (EPS) layer of the Biofilm makes the cell resistant to antibiotics by means of hindering its action upon the organism (Shankar et al. 2003; Mittal et al. 2015). Therefore, the immediate and alternate antibiofilm agents are required to combat the disease caused by biofilm forming *E. coli*. The emergence of resistance genes within the biofilm has made biofilmers resistant to antibiotic treatments. Therefore, there is a crucial need to eradicate biofilm forming *E. coli* by means of alternative therapeutic strategies such as nanoparticles (Roy et al. 2018).

Phytopathogenic fungi causes drastic threat to the agriculture since ancient days. Fungal infection causes enormous impact towards the socio-economic development of the country. Among the fungal species *Aspergillus niger, Fusarium udum, Fusarium graminearum* cause diseases to plants and affect agriculture in terms of affecting the plant growth, yield and storage. *A. niger* is a plant pathogen which causes spoiling of numerous fruits and vegetables throughout the world. Among which *A. niger* tremendously affect the storage of onion by causing black rot disease, storage of pomegranate by causing black spot diseases. Apart from infecting plants, *A. niger* infects human by infecting the tissues and bronchus by causing aspergillosis. *A. niger* produces toxic compound such as mycotoxins and ochratoxins. These toxins not only affect human but also other plants and vertebrates. *Fusarium* head blight (FHB), in barley and wheat is caused by *Fusarium graminearum*. It is one of the important cereal crop diseases all over the world, which affects the yield of the crop by reducing the quantity of gluten and albumin during harvest. The harvested cereal is also contaminated with deadly trichothecene mycotoxins making the food and feed unsafe to humans and animals. Sesquiterpenoid mycotoxins produced by *F. graminearum* are DON, 15A-DON and 3A-DON, (DON-Deoxynivalenol) are responsible for the virulence, which inhibits translation in eukaryotes. *Fusarium udum* is the soil borne pathogen, causative agent of a wilt disease on pigeon pea, ear rot disease, seedling blight, reduction in seed germination, severe reduction in quality and yield of small cereals including maize, oat, barley and wheat, which causes significant economic loss all over the world. *F. udum* produces fusaproliferin (FUS)-bicyclic sesterterpene mycotoxins, contaminating the cereals, which impact on animal and human health (Dean et al. 2012).

In recent years, Breast Cancer has been known to reflect a significant rate of mortality among women. There is a lack of evidence on therapeutic paradigm for regulation and possible treatment against breast cancers (Rohini et al. 2019). Human breast cancer MCF-7 cells are well chosen experimental model all over the globe (Shariq et al. 2019). No reports published on the targeted therapy of CANCs against breast cancer in an in vitro and in vivo system.

In this study, we have focussed on the synthesis of aromatic nanocolloids (CANCs) using citronella extract. CANCs were tested against biofilm forming pathogenic *E. coli*. The antibacterial activities of the synthesized CANCs were evaluated through Minimal inhibitory concentration, Minimum bactericidal concentration, time course experiments and biofilm inhibition assay and analysis of gene encoding CTX-M-15 which is involved in Extended-spectrum beta-lactamases (ESBL) production (Peirano et al. 2010; Rasool et al. 2018). Therefore, CTX-M-15encoding pathogenic, biofilm forming *E. coli* are used as test organism for this research to explore the antibacterial action of CANCs. The efficacy of this CANCs in controlling plant pathogenic fungi was screened. Additionally, the cytotoxic potential of CANCs was tested in MCF-7 cells under in vitro conditions.

## Materials and methods

Test organism used in this study was obtained from Tagore Medical College and Hospital, Chennai after proper ethical approval (Ref. no. BSAU: REG-OFF: 2016/02SLS). The strains used for this study are *E. coli* ATCC (25922), *F. graminearum* MTCC (2089), *F. udum* MTCC (2204), *A. niger* MTCC (281). MDR *E. coli* was isolated from Urinary tract infected sample. Human breast cancer cell lines MCF-7 were procured from National Centre for Cell Science (NCCS), Pune, India.

### Preparation of citronella extract

The aromatic extract of citronella was prepared through following steps. 10 g of powdered citronella grass was mixed with 100 ml of sterile water. Decoction of citronella was prepared by boiling to obtain concentrated syrup. Concentrated syrup was subjected to centrifugation at 1000 rpm for 5 min to remove larger particles. It was followed by filtering through grade1 filter paper thrice to clarify the syrup. Then, the concentrated syrup was stored at 4 °C for further use (Ranjani et al. 2019a). Qualitative phytochemical screening of citronella grass extract was carried out using standard methods (Harborne 1973).

### Synthesis of Citronella extract mediated aromatic nanocolloids (CANCs)

CANCs were synthesised using 1 mM silver nitrate solution. The filtered aqueous extracts of citronella were mixed with 1 mM AgNO_3_ solution in the ratio of 1:2. The flasks were micro irradiated for 2 min for nanocolloid synthesis. The formation of silver nanocolloids was observed through visible colour change and it was confirmed through UV–visible spectroscopy. Then the synthesised nanocolloids were centrifuged at maximum RCF for 15 min and the settled nanoparticles were suspended in autoclaved distilled water and centrifuged again at maximum RCF (Akther et al. 2018). The resulting pellet was suspended in 20% DMSO by means of sonicating the colloidal suspension for 5 min. This colloidal suspension was used for characterisation and for evaluating its toxicity, antibacterial, antibiofilm, anticancer and antifungal activities.

#### Characterization CANCs

UV–visible spectroscopy was used for confirming the synthesis of CANCs, by scanning between the range of 200–800 nm. The footprint of functional groups present in the synthesized nanoparticles were analysed through FT-IR spectroscopy, where both the aqueous extracts and CANCs are used and scanned between the range 4000–400 cm^− 1^ (Perkin Elmer Spectrum100) (Akther et al. 2019). The synthesised CANCs was analysed through particle size analyzer (Malvern Instruments Ltd), to calculate the average diameter of CANCs based on the random movement of particles present in the colloidal solution. Analysis of synthesised CANCs was carried under the surrounding medium viscosity of 0.8872cP, count rate of 273.1 kCPS, at 25 °C. The zeta values help us to find out the stability of CANCs by measuring the surface charge distribution around the nanocolloid (Malvern Instruments Ltd) (Kaviya et al. 2011). Field Emission Scanning Electron Microscope (FESEM) was carried out to study the morphology of CANCs. Energy Dispersive X-Ray Analysis (EDAX) was performed to confirm the elemental composition of CANCs (SIGMA HV-Carl Zeiss with Bruker Quantax 200-Z10 EDS Detector).

#### Toxicity assay of green nanocolloids in buffalo oocyte maturation

Collection, retrieval, grading of cumulus oocyte complexes (COCs) was achieved as per the standard procedure (Nandi et al. 1998). To check the toxicity of CANcs, the medium was added with 1 µl (1 mg/ml) of CANCs. Maturation assay was carried out by protocol of Ravindranatha et al., with some modification (Ravindranatha et al. 2002). The degree 1 and 2 oocyte with cumulus expansion were considered as matured.

#### Assessment of antimicrobial activity and antibiofilm activity

In our antimicrobial study, MIC and MBC was carried out with test strains as *E. coli* ATCC (25922) and three different biofilm forming clinical strains of pathogenic *E. coli*. MIC was carried to calculate the minimum inhibitory concentration of CANCs, using Broth dilution method and microtiter plates were read at 600 nm using Microtiter plate reader (Perkin Elmer Inspire, Multimode reader) (Ranjani et al. 2019a,b; Sah et al. 2018). MBC was carried out using drop plate method to calculate the minimum bactericidal concentration (Xiang et al. 2019). Biofilm inhibition assay was carried using microtiter plate method, to calculate the concentration of CANCs which completely inhibit the biofilm formation. The formation of biofilm was calculated spectrophotometrically by measuring at A580, using a microplate reader (Perkin Elmer Inspire, Multimode reader). From this measurement we can quantify the concentration of CANCs, required to inhibit the biofilm formation in in vitro condition (Ranjani et al. 2019a, b; Akther et al. 2018).

#### CANCs modulate the expression of CTX-M-15 and AmpC genes

The expression of CTX-M-15 and AmpC genes was assessed by gene amplification in control and CANCs treated strains. Polymerase Chain Reaction was carried out for following set of gene specific primers CTX-M-15 and AmpC genes as described in Ranjani et al. (2019a, b) without any modification (Ranjani et al. 2019a; Ranjani et al. 2019b; Sah et al. 2018).

#### Antifungal activity of CANCs

50 ml of potato dextrose agar plates were prepared with two different concentrations (10 µg/ml and 20 µg/ml) of CANCS. The control plates were prepared without CANCs. *A. niger* MTCC (281), *F. udum* MTCC (2204), *Fusarium graminearum* MTCC (2089) inoculated in green nanocolloid supplemented plates (10 µg/ml and 20 µg/ml) and in control plates, using hyphal tip method. The plates were incubated at 28 °C for 5 days with periodic monitoring for the fungal growth. After 5 days the diameter of fungal growth in each of the treated plates (10 µg/ml and 20 µg/ml) were measured when the control fungal group reached its growth of 5 cm. The growth of fungal strain was continuously measured at the intervals of 3 days. The percentage of inhibition was calculated using the formula:

$$Percentage\,of\,inhibition = [D]-[d] [D] \times 100.$$

where ‘D’ is the diameter of the fungal growth in control group, and ‘d’ is the diameter of the fungal growth in CANCs treatment group (Shunyu et al. 2019).

### Anticancer effect of CANCs

#### Treatment regimen

The assays were planned in such a way, at 24–48 h time points, MCF-7 cells were seeded in culture plates. Synthesized CANCs were dissolved in DMSO to obtain 1 mg/ml stock solution, which was further diluted appropriately at the time of exposure (Rohini et al. 2019). To induce cytotoxicity, MCF-7 cells were treated for 24 h with different concentrations of CANC’s (0–100 µg/ml) under in vitro conditions. Each experiment was repeated at least 2–3 times. For investigation of synthesized CANCs induced cytotoxicity, cells were divided into six groups: group I (control, cells treated with DMSO and considered as 0 µg /ml), group II (cells treated with 1 µg/ml CANCs), group III (cells treated with 5 µg/ml CANCs), group IV (cells treated with 10 µg/ml CANCs), group V (cells treated with 50 µg/ml CANCs), and group VI (cells treated with 100 µg/ml CANCs). At the end of the exposure, MTT and LDH Assays were performed.

#### Cytotoxicity assay

Cytotoxicity was measured based on the conversion of MTT to formazan crystals by mitochondrial dehydrogenases (Shariq et al. 2019; Waseem et al. 2017). Briefly, 2 × 10^3^ cells per well were seeded on poly-l-lysine pre-coated 96 well culture plates and allowed to adhere for 24 h in 5% CO_2_ incubator at 37 °C. After 24 h, the cells were then incubated with CANCs (0–100 µg/ ml) for 24 h in their respective media. Thereafter, half of the media were replaced with serum-free DMEM containing 0.5 mg/ml MTT and incubated at 37 °C in a CO_2_ incubator for an additional 1 h. Intracellular formazan products were solubilized by replacing the MTT reagent with MTT solvent (4 mM HCl and 0.1% NP-40 in isopropanol) and incubated for 15 min at 37 °C under 5% CO_2_. Contents were transferred to a 96-well plate and optical density (OD) was measured at 570 nm with reference wavelength at 620 nm using Gen 5.0 software provided with the plate reader (Power Wave XS2; BioTek, Winooski, VT). Only MTT solvent was used as reference. Each experiment was performed in triplicate and repeated three times for cultured cells. The cytotoxicity results are expressed as the percentage of toxicity compared to the control.

#### LDH release assay

The permeability of cell membrane in experimental MCF-7 cells were investigated by LDH release assay (Waseem et al. 2017). Briefly, 2 × 10^3^ cells per well were seeded on poly-l-lysine precoated 96 well plates and allowed to adhere for 24 h in 5% CO_2_ incubator at 37 °C. After 24 h, the cells were then incubated with CANCs (0–100 µg/ ml) for 24 h in their respective media. An aliquot of 4 mM of pyruvic acid in 100 mM of PBS (pH 7.5) was added to the plate containing 0.4 µg/µl β NADH in 100 mM PBS at the pH of 7.5. The LDH release was quantified as percentage of LDH in medium as compared to total LDH in lysates of MCF-7 cells (Bergmeyer and Bernt 1974).

## Results

### Synthesis and physio chemical characterisation of CANCs

**Fig. 1:**
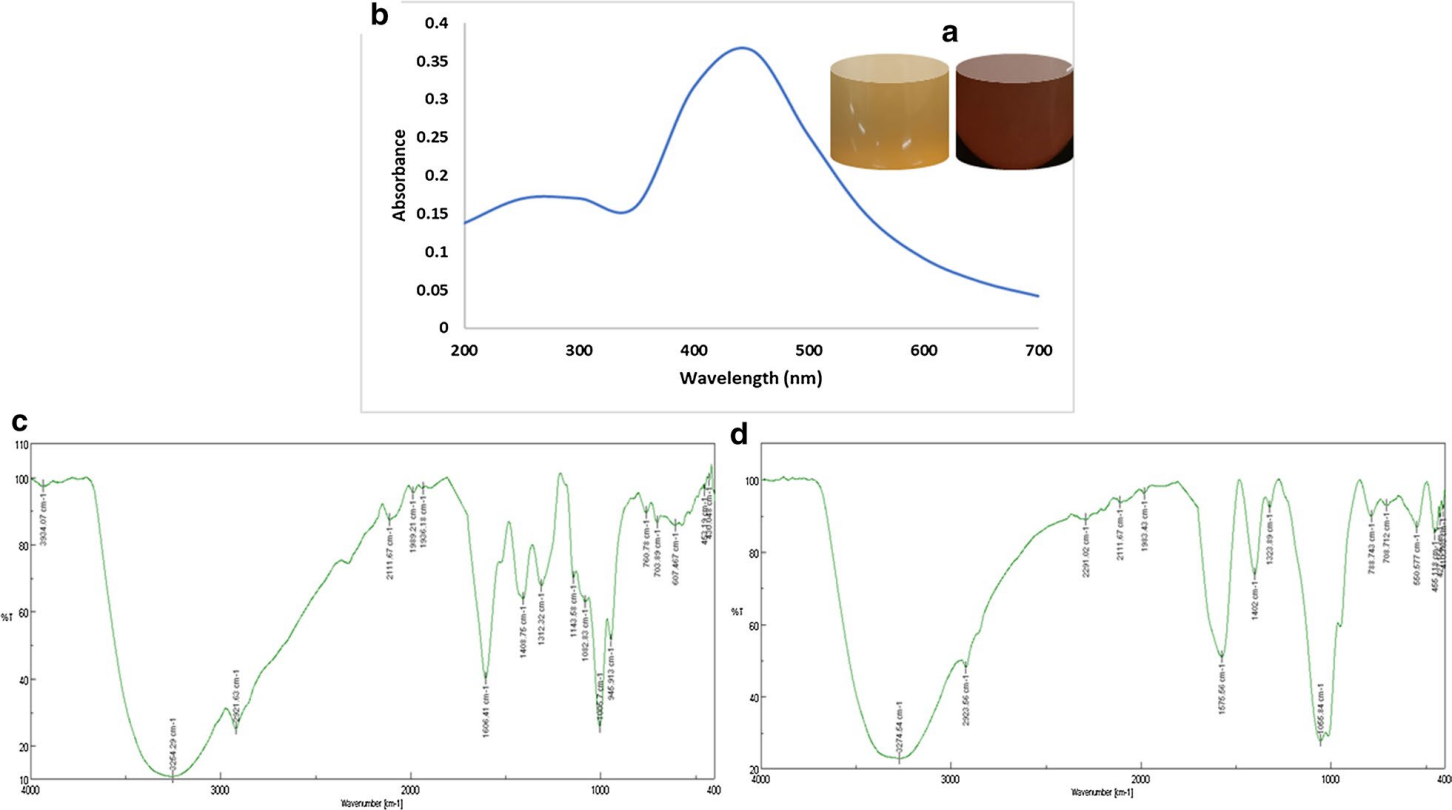
Synthesis and characterization of the CANCs. **a** Colour change of the citronella extract during CANCs synthesis. **b** Absorption spectrum of CANCs nanocolloids. **c** FT-IR spectrum of Crude citronella extract. **d** FT-IR spectrum of CANCs

Citronella extract was prepared and subjected to phytochemical analysis. The results of phytochemical analysis of citronella extracts are shown in Table 1. To 100 ml of citronella syrup, 200 ml of 1 mM silver nitrate was added and mixed well. The solution was micro-irradiated for 5 minutes and the colour change was observed immediately which was the preliminary indicator for the synthesis of CANCs. The visual colour change of CANCs is depicted in figure (Inlet Fig. 1a). Further CANCs synthesis was confirmed through UV–Visible spectroscopy by scanning the absorption spectra between 200 and 800 nm. The observed surface plasmon resonance peak confirmed the synthesis of CANCs (Fig. 1b).

**Table 1:** Phytochemical analysis of citronella extract

Extract	Phenol	Tannins	Flavonoids	Saponins	Anthocyanins	Terpenoids	Alkaloids	Steroids	Carotenoids
Citronella extract	+	+	+	+	−	+	−	+	−

FTIR spectra was taken for both crude extract and CANCs. The extracts and CANCs were found to have aromatic compound, aldehyde, Alkene, flourocompound, which were confirmed by showing strong peak at their respective wave number (Fig. 1c, d), The details of wave number and corresponding functional groups are shown in Table 2.

**Table 2:** FT-IR spectral analysis of the compounds present in crude extract and CANCs

Name of the extract/Ncs	Frequency (/cm)	Bond	Functional group
Citronella extract	3339.14	$\equiv$C$-$H stretch	Aromatic compound
	2920.66	$-\text{C}-\text{H}$	Aldehyde
	1031.59	C$-\text{F}$	Fluro compound
CANCs	3274.54	$\equiv$C$-$H stretch	Aromatic compound
	2923.66	$-\text{C}-\text{H}$	Aldehyde
	1055.84	C$-\text{F}$	Fluro compound

**Fig. 2:**
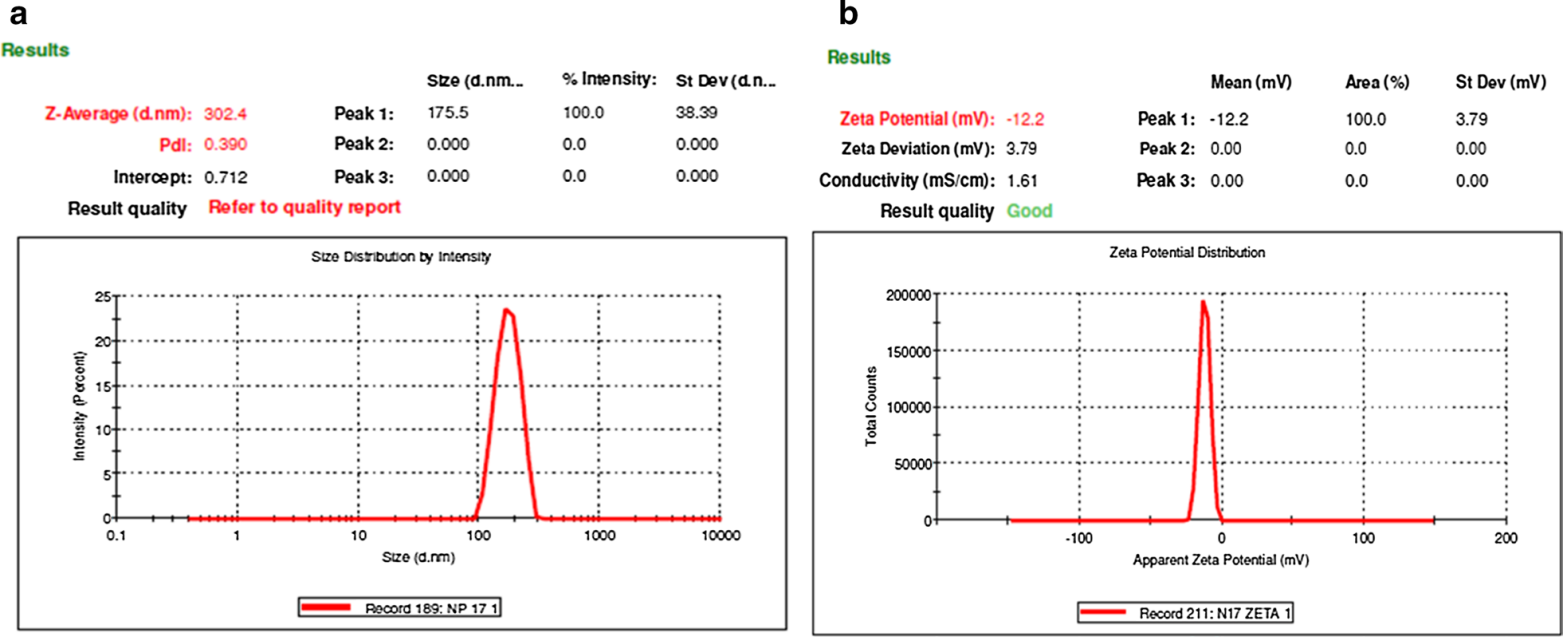
**a** Particle size analysis of CANCs, **b** Analysis of Zeta potential of CANCs

The hydrodynamic size, PDI, and surface charge of CANCs were calculated using the dynamic light-scattering (DLS) technique (Fig. 2a). The size of CANCs was found as 302.4 nm. PDI is dimension less and scaled by the value, PDI value “0” indicates that nanoparticles are monodispersed in solution, value “1” indicates polydispersity of nanoparticles in solution. The PDI of CANCs was found as 0.390. CANCs had mid-range value, and all are in acceptable range. The presence of the minor and major peaks could have been due to polydispersity in the size or shape of the nanoparticles (Fig. 2a) (Premasudha et al. 2015).

The zeta potential of CANCs was observed as − 10.4 (Fig. 2b). The results showed that the charges distributed on the surface of nanoparticles present in CANCs was negatively charged. In addition, nanoparticles are polydispersed in the colloidal medium. The negative charge introduces repulsion among the nanoparticles and prevent the aggregation of particles present in the colloidal solution. This provides the stability of the colloidal solution for long term storage even at room temperature (Anandalakshmi et al. 2015).

**Fig. 3:**
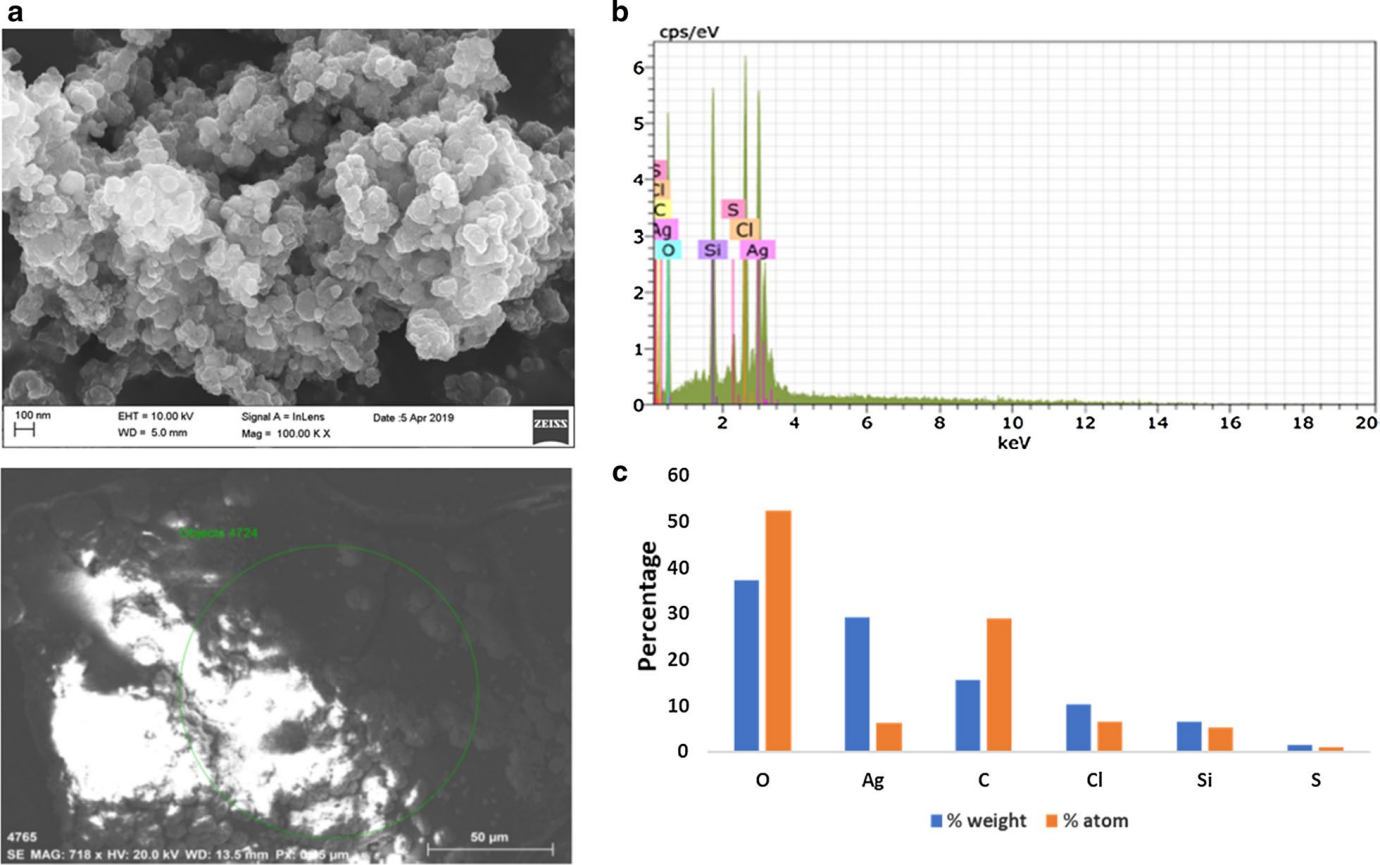
**a** FESEM imaging of CANCs, **b** EDAX analysis of CANCs, **c** Percentage of distribution of atoms in CANCs

The topography of the CANCs were obtained using Field Emission Scanning Electron Microscopy (FESEM). The FESEM image of CANCs were spherical in shape at 200 nm resolution under the magnification of 50.00KX (Fig. 3a). EDAX analysis is used for the elemental information present in the CANCs. The EDAX results confirmed the presence of silver, by showing strong elemental peak at 3 kev (Fig. 3b). It was reported that silver nanoparticles show optical absorption peak approximately at 3 keV because of their surface plasmon resonance. The percentage weight and percentage atom of CANCs were obtained from EDAX results. The result showed that, weight percentage and atom percentage of Ag was high in the aromatic nanocolloids (Fig. 3c).

### Toxicity study of green nanocolloids on Buffalo oocyte maturation

**Fig. 4:**
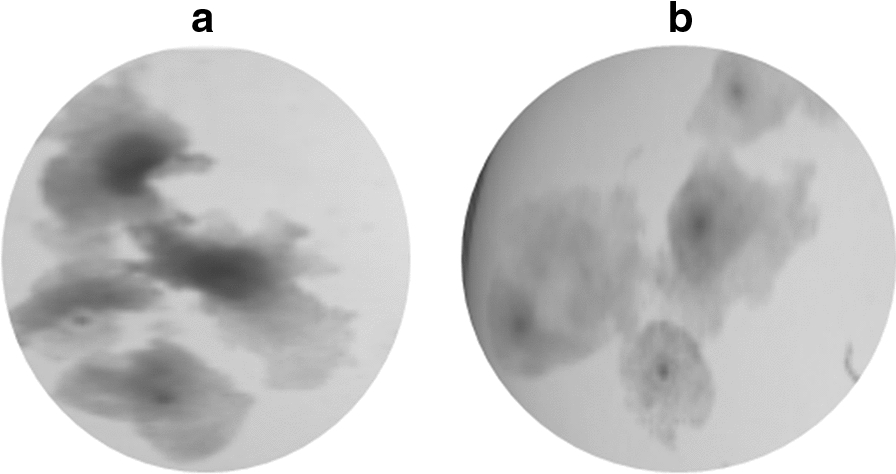
Toxicity studies of CANCs on oocyte maturation under stereo zoom microscope in 10× magnification. **a** Oocyte maturation in IVM medium as control, **b** Oocyte maturation in IVM medium treated with CANCs

Collection, retrieval, grading of cumulus oocyte complexes (COCs) was carried out as described in the methods. The buffalo COCs graded based on their homogeneity of ooplasm and cellular investment. The percentage of oocyte maturation under CANCs was found to be 95.2%, when compared to control. The microscopic examination of oocyte maturation was observed under fluorescence microscope under magnification of 10× which depicts that CANCs treatment did not affect the oocyte maturation (Fig. 4a, b).

### Assessment of antibacterial activity of nanoparticles through MIC, MBC and biofilm assay

**Fig. 5:**
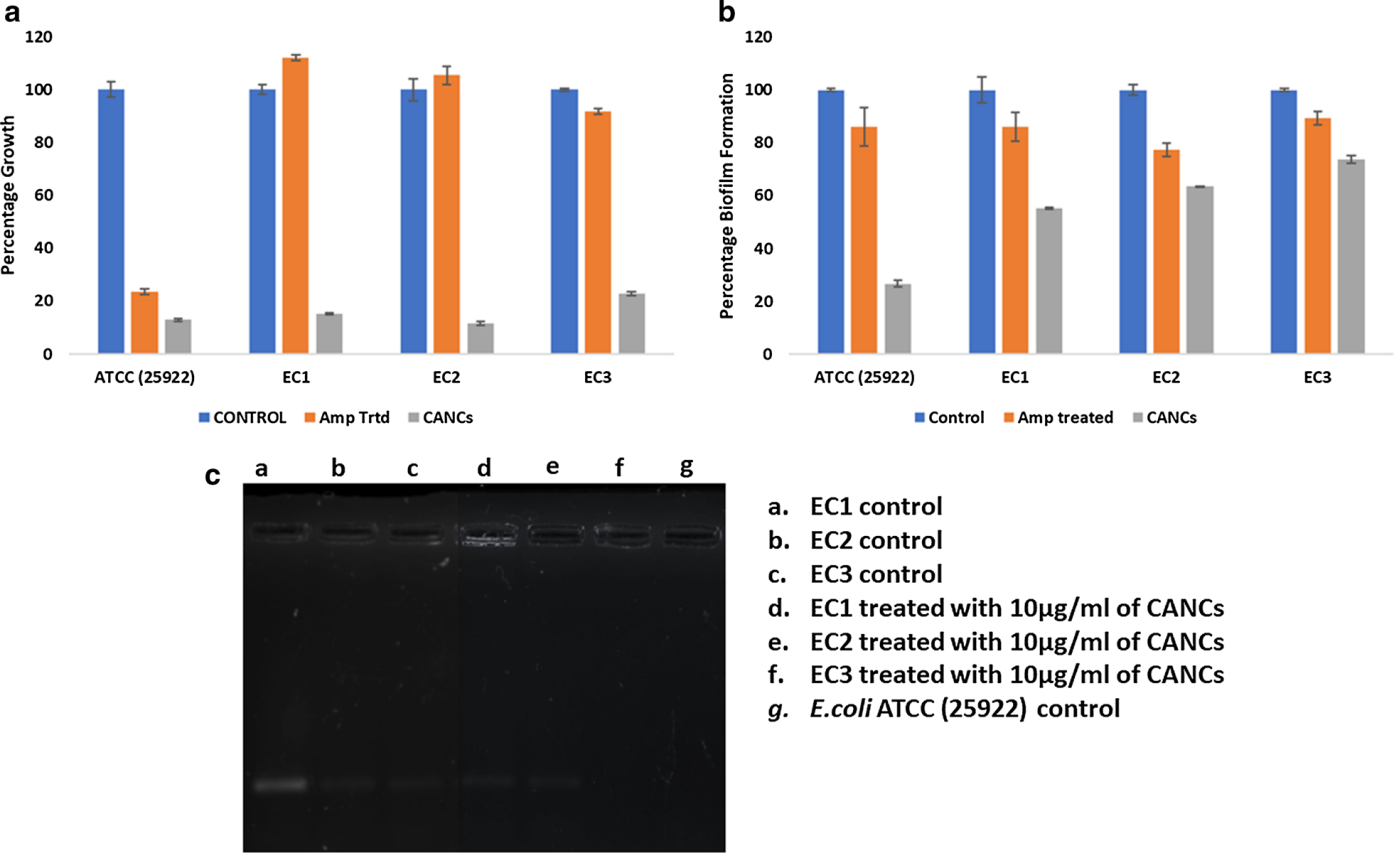
** a** Analysis of growth percentage in control and treated with antibiotic and CANCs in ATCC (25922) and clinical strains of *E. coli*. **b** Analysis of biofilm formation in control, treated with antibiotics and CANCs in ATCC (25922) and clinical strains of *E. coli.* **c** Analysis of expression of CTX-M-15 gene in control and treated with CANCs; (**a** EC1, **b** EC2, **c** EC3 control) (**d** EC1, **e** EC2, **f** EC3 treated with CANCs), **g** ATCC (25922) Control

The Minimum inhibitory concentration of CANCs was validated against *E. coli* ATCC (2922) and three biofilm forming pathogenic *E. coli* (EC1, EC2, EC3). The bacteriostatic effect of CANCs towards *E. coli* ATCC (25922) and three biofilm forming pathogenic *E. coli* (EC1, EC2, EC3) were different from each other. The results showed that CANCs inhibited the growth of all test strains (ATCC (25922), EC1, EC2, EC3) at the concentration of 1 µg/ml. The growth rate was reduced up to 13% ,15%,12%, 23% for ATCC (25922), EC1, EC2, EC3 strains respectively, when compared to ampicillin treatment and control (Fig. 5a). Similarly, the minimal bactericidal concentration was calculated to find out the bactericidal concentration of CANCs which completely killed the organism. MBC results for CANCs against the ATCC (25922), EC1, EC2, EC3 *E. coli* strains are shown in Table 3. Bactericidal concentration of CANCs was found to be 1.0 µg/ml, 1.5 µg/ml, 1.5 µg/ml, 1.0 µg/ml for ATCC (25922), EC1, EC2, EC3 *E. coli* strains respectively. The results from biofilm assay confirms that minimum 1.5 µg/ml of CANCs was required to act as anti-biofilm agent in all tested *E. coli* strains. Upon CANCs treatment the biofilm formation was inhibited by 66%, 49%, 59%, and 50% in ATCC (25922), EC1, EC2, and EC3 *E. coli* strains respectively (Fig. 5b).

**Table 3:** Determination of minimum inhibitory concentration (MIC) and minimum bactericidal concentration (MBC) of the CANCs treated

S. no.	CANCs (µg/ml)	
	MIC	MBC
*E. coli* ATCC (25922)	1.0	1.0
EC1	1.0	1.5
EC2	1.0	1.5
EC3	1.0	1.0

### CTX-M-15 and AmpC gene expression studies

In the current study, the expression of antibiotic resistance gene CTXM-15 and AmpC genes was analysed in ATCC (25922), EC1, EC2, and EC3 *E. coli* strains. Amplification was carried out from both treated and untreated strains. From the result it was observed that, upon treatment with CANCs the expression of CTXM-15 gene was down regulated in EC1 and EC2 strains and it was completely abolished in EC3 strain (Fig. 5c).

Further in *E. coli* strains expression of AmpC gene was analyzed. The results showed that AmpC gene was absent in these test organisms, suggested that these strains may follow the CTXM-15 or any other antibiotic resistance genes pathway to break antibiotic resistance.

### Antifungal activity of green nanocolloids

**Fig. 6:**
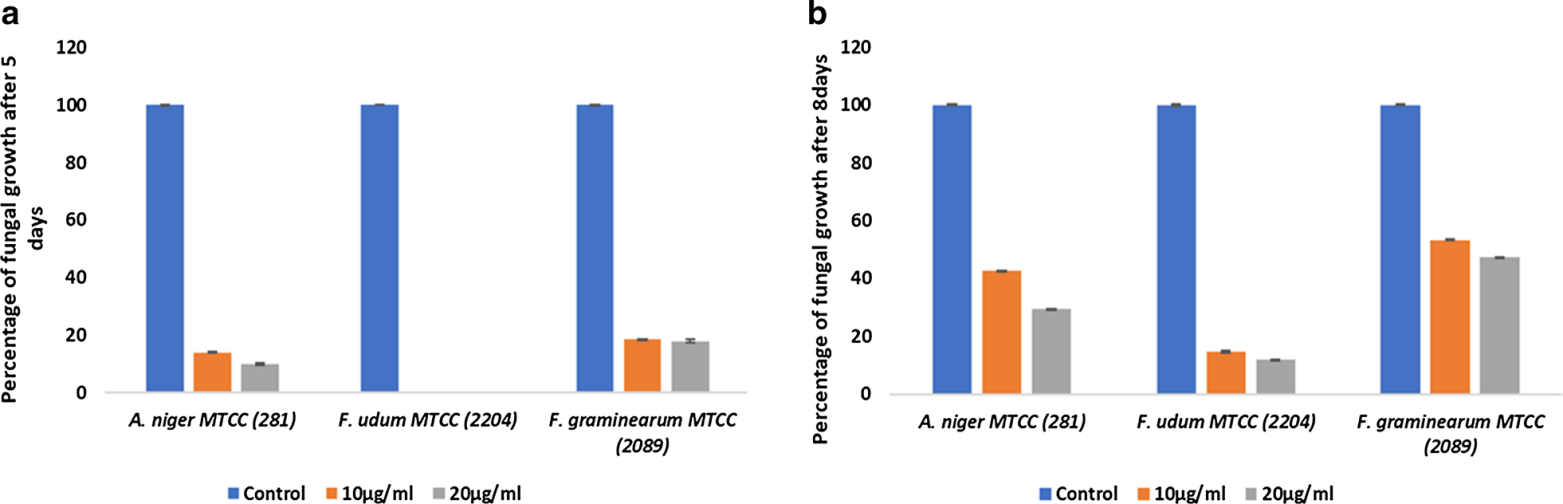
">**a** Percentage growth of fungi after 5 days of incubation, treated with 10 µg/ml and 10 µg/ml of CANCs. **b** Percentage Growth of fungi after 8 days of incubation, treated with 10 µg/ml and 10 µg/ml of CANCs

CANCs showed potent antifungal activity against all three tested plant pathogenic fungi. Fungal strains were inoculated using hyphal tip method in control and CANCs treated plates at the concentration of 10 µg/ml and 20 µg/ml. Fungal growth was observed after 5 days of incubation at 28 °C. In control plate the growth of all the three fungi reached the full plate of 5 cm, whereas *A. niger* MTCC (281) started its sporulation, *F. udum* MTCC (2204) and *F. graminearum* MTCC (2089) started spreading its hyphae throughout the plates. In 10 µg/ml CANCs treated plate there was reduction in growth rate of fungi when compared with the control. The reduction in growth rate was found to be 86%, 100%, 81% for *A. niger* MTCC (281), *F. udum* MTCC (2204) and *F. graminearum* MTCC (2089) respectively (Fig. 6a). In 20 µg/ml CANCs treated plate the reduction in growth rate was found to be 97%, 100%, 88% for *A. niger* MTCC (281), *F. udum* MTCC (2204) and *F. graminearum* respectively (Fig. 6a).

Further the results were extrapolated by monitoring the fungal growth for upto 8 days of incubation at 28 °C. The fungal growth was measured for 10 µg/ml of CANCs treated plate and it was observed as 57%, 85% and 47% for *A. niger* MTCC (281), *F. udum* MTCC (2204) and *F. graminearum* MTCC (2089) respectively, when compared with the control (Fig. 6b). In 20 µg/ml of CANCs treated plate the inhibition rate was observed to be 70%, 88% and 52% for *A. niger* MTCC (281), *F. udum* MTCC (2204) and *F. graminearum* MTCC (2089) respectively (Fig. 6b).

### Anticancer activity of CANCs

**Fig. 7:**
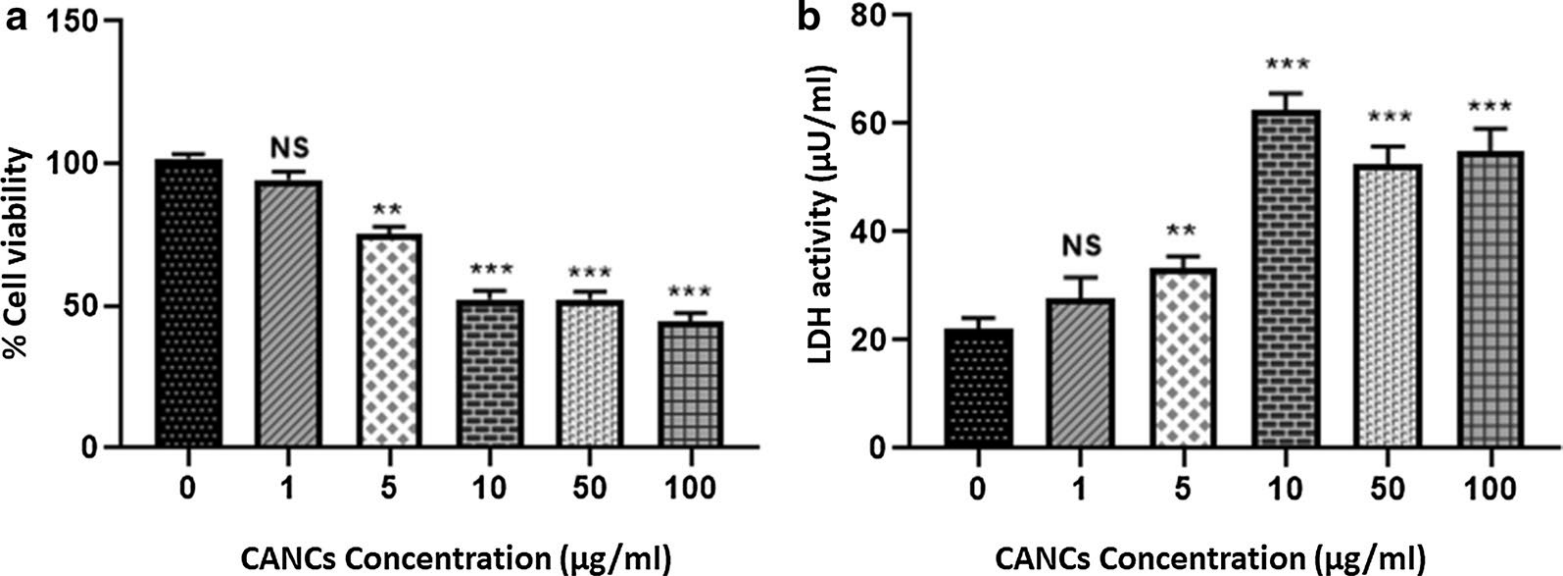
Anticancer activity of CANCs: **a** Effect of CANCs on cell viability of MCF-7 cells treated with various concentrations of CANCs. **b** Shows LDH based cytotoxicity assay in MCF-7 cells

Cytotoxicity via CANCs treatment was determined by using MTT reduction assays in experimental cells. As shown in Fig. 7a, cell viability was significantly decreased in a dose dependent manner (P < 0.01–P < 0.001) in MCF-7 cells when compared to control. In addition, LDH release was also evaluated in MCF-7 cells exposed to CANCs. Since, LDH release is a well prominent marker for an exact measurement of the cellular membrane integrity and cell viability. Figure 7b shows the significant alteration (P < 0.01–P < 0.001) in LDH activity as compared to control. Minimal dose of CANCs at 1 µM did not show any significant change in LDH activity of the experimental cells.

## Discussion

Green nanocolloids play an important role in cancer diagnosis, antibacterial activity, antimicrobial, anticancer, antifungal and antiviral etc., (Iravani et al. 2014). The green nanotechnology paved the way for the development of eco-friendly nano-based solution to plants, animals and human beings. Herein we developed green silver nanocolloid using citronella extract. Citronella contains various phytocompounds such as steroids, phenols, tannins, alkaloids, flavonoids, phytosteroids, glycosides, which could be harnessed for its antibacterial, antifungal and anticancer activity. These phytochemicals of Citronella paved the way to explore more on drug development and its action on treating several diseases. The phytochemical tests are also consistent with previous literatures through confirming the presence of bioactive compounds such as alkaloids, anthocyanins, carotenoids, flavonoids, phenols, tannins, terpenoids, saponins and steroids. Plant phenols possess antioxidant activity which helps in reducing free radicals and it interferes with all stages and types of cancer due to its antioxidant activity. Flavonoids can inhibit the formation of peroxides. Phenols, Tannins, flavones, flavonoids, alkaloids, terpenoids and saponins possess significant antibacterial, antiviral, anti-inflammatory and anti-tumor properties (Shashank et al. 2013).

Biogenesis of CANCs was physio-chemically characterized by UV Vis spectrum, FTIR, PSA, DLS, FESM, EDAX. By observing the UV Vis spectrum SPR peak confirmed the synthesis of CANCs. It was reported that the peak absorbance of silver nanoparticles produced through microwave irradiation is higher than that of room temperature reaction (Abdalla et al. 2014). It was reported that thermal conditions modify the size and shape of synthesised nanoparticles (Raut Rajesh et al. 2009) and temperature increases the biogenesis of silver nanoparticles (Kanchana et al. 2010).

Phytocompound enriched citronella extract acted as an excellent reducing agent for the synthesis of CANCs. This triggered the conversion of Ag + to AgO which occurs during the formation of enol/keto form of those phytocompounds (Siemieniec 2013). Citronella is rich in terpenoids, which was reported to play a key role in reduction of silver ions (Mittal et al. 2015). Flavonoids, proteins, polysaccharides are reported to bind to the surface of nanoparticles (Sathishkumar et al. 2009). Water soluble phytocompounds flavonoids and terpenoids played crucial role in the reduction of silver ions upon microwave irradiation. The FTIR spectra was used to find the potential bioactive compounds which are responsible for the capping and reduction during the synthesis of CANCs (Ranjani et al. 2019a). Thus, the FTIR results revealed the mixture of phytocompounds present in the extract assist the reduction and stabilization of CANCS synthesis simultaneously. The size and stability of colloidal nanoparticles was measured using the Brownian movement of the nanoparticles shows CANCs were in nanometer in range and stable without aggregation. FESEM EDAX showed the presence of other elements C, O, Si, Cl, S could be derived from the bioactive compounds which were in the citronella extract, which may be bound to the topical surface of silver nanocolloids, which may played the role in bioreduction (Ndikau et al. 2017). Apart from Ag, elements such as C, Cl, Si, S has potent antibacterial activity. These elements work synergistically in imparting antibacterial, antifungal and anticancer activity (Abdalla et.al. 2017). Toxicity study of CANCs on Buffalo oocyte maturation confirmed its non-toxicity even at the reproductive and embryonic developmental stages. Upon treatment it indicates the occurrence of complete maturation and readiness of the oocytes to undergo fertilization.

In this present study CANCs were found to be very efficient antibacterial activity, which was confirmed through bacteriostatic (MIC) and bactericidal (MBC) assays. The phyto constituents present in the citronella extract possess good antibacterial activity along with the Ag, Si, S, Cl. It was reported that Silver nanoparticles exert their antibacterial effects by attaching to the membrane and modulating cellular signalling pathways by means of penetrating inside the cell. The main mechanism by which silver nanoparticles exhibit antibacterial properties is by means of dephosphorylating the trypsin residues. After penetration into the bacteria, silver nanoparticles inactivate the enzymes, which stop many of the signalling pathways and metabolic pathways, eventually lead to bacterial cell death (Fatem et al. 2011). Previous studies reported that Citronella nanoparticles possess antibacterial activity against multiple drug resistant hospital isolates of *E. coli*, *S. typhi, P. mirabilis K .pnuemoniae, S.aureus*. There was also report on the green silver nanoparticles, which effectively control the growth of *Escherichia coli, Pseudomonas aeruginosa, Staphylococcus aureus* and *Bacillus cereus* (Saliem et al. 2016). CANCs showed effective biofilm inhibition activity, which could be due to its better penetrating ability of nanocolloids into the slimy layer of biofilm and successfully reduced the formation of biofilm by controlling the bacterial growth. The shape, size, and surface charge of CANCs play critical role in penetrating into the biofilm layer and kill the organism by anyone of the mechanism such as ROS production, cell wall damage, DNA damage etc. In previous studies it was reported that polyphenolic compounds, tannins, terpenoids, flavonoids, which are present in the plant extracts, acted as a siderophores, which chelates the metal from the growth medium and degrades the biofilm (Sharma et al. 2016).Similar results were supporting in our study, in such a way that citronella extracts were rich in polyphenols, tannins, terpenoids, flavonoids which acted as a reducing agent during nanocolloid synthesis, which reacted as quorum quenchers by disturbing the biofilm formation by preventing the cell to cell communication (Qing et al. 2018).

From the result of CTX-M-15 gene expression studies, it can be hypothesized that CANCs suppress CTX-M-15 gene expression which in turn reduce the transcription and translation of enzyme which is responsible for antibiotic degradation. There were previous report supporting that silver ions directly interact with DNA, RNA and protein, which disturb the central dogma of the cell (Qing et al. 2018). Thus, reproductive mechanism of the organism will be prevented, which ultimately reduces the pathogenicity of microorganism through various means such biofilm inhibitor, suppressing the antibiotic resistant genes etc., (Peirano et.al. 2010).

The antifungal activity of CANCs was validated against three different plant pathogenic fungi *A. niger MTCC (281)*, *Fusarium udum* MTCC (2204), *Fusarium graminearum* MTCC (2089). This is the first report to study the antifungal activity of CANCs. All these plant pathogenic fungi cause severe damages to crop, cereals, fruits and vegetables in course of pre and post harvesting and greater economic loss to the country. This study confirmed that CANCs is a potent broad-spectrum antifungal agent. The difference in the inhibition rate was observed because each fungus differs in their growth rate by the physiological changes induced by the CANCs. There are few literatures supporting that the lemon grass oil has potent antifungal activity, CANCs with the synergistic effect of both silver and phytocompounds played a crucial role in inhibiting the fungal growth. There are few literatures supporting that silver nanoparticles affect the morphology of mycelium and affect the normal growth of hyphae. Green nanoparticles attach to the cell membrane, which in turn damages the cell membrane and changes the permeability of the cell wall. By altering the membrane permeability, the CANCs can easily enters the cell, disturbs the normal metabolism, and damages the cell organelles. Previous studies showed that silver nanoparticles damage the cell membrane, reduce the enzyme activity and affect the replication, transcription and translation process (Shunyu et al. 2019).

CANCs induced apoptosis via enhancing the release of lactate dehydrogenase enzyme in MCF-7 cells. MTT assay is an important process to recognize the activity of NAD(P)H dependent oxido-reductase enzyme that are capable to convert MTT to formazan in viable cells, on the contrary, LDH measures the released LDH enzyme from damaged plasma membrane of the cells. Similarly, SRB assay measures the amount of protein present in viable cells. Therefore, the therapeutic interventions of CANCs encapsulated molecules might be challenging chemoprevention candidate, with its ameliorative efficacy in cancers, including breast cancer (Shariq et al. 2019).

In this study, we adopted new strategy of green nanocolloid synthesis using Citronella extract by means of microwave irradiation. The microwave irradiation method was found to be rapid and efficient approach involving environmentally friendly and using low cost reductant flora, besides using toxic chemicals, for synthesizing CANCs. CANCs showed effective antimicrobial, biofilm quenching agents on biofilm forming *E. coli.* Further CTX-M-15 gene expression studies showed effective down regulation of CTX-M-15 gene in clinical isolate upon treatment with CANCs. These CANCs may be formulated as different types of nanoformulations such as nanogel, nanospary to fight against these deadly pathogens. Further, CANCs was validated against three phytopathogenic fungi *A. niger MTCC (281), F. udum* MTCC (2204), *F. graminearum* MTCC (2089) and confirmed it as effective antifungal agent. It was found that CANCs significantly reduces the growth rate of fungi through affecting morphology of hyphae. Hence, CANCs could be an effective alternative to chemical fungicides. This CANCs can be formulated as nano spray in effective way of controlling fungal infection in agricultural farming of cereals, fruits, vegetables and in long term storage of food commodities. CANCs can be used to eliminate the contamination of food grains with mycotoxins and protect the living beings from associated health effects. In addition, Cytotoxic activities of CANCs in MCF-7 cells revealed the efficacy of CANCs in decreasing the cell viability and can be used in formulating anticancer drugs at sub cellular, molecular and translational studies, however further in vivo studies are warranted. Further studies on optimisation of CANCs in different nanoformulations will help us in mass production and commercialization for various applications.

## Data Availability

Data will be available on request.
